# The Metadynamic Recrystallization Role in Ultrafast <111> Fiber Texture Evolution During Short-Term Holding in β-Forged Ti-6242

**DOI:** 10.3390/ma18194447

**Published:** 2025-09-23

**Authors:** Haodong Rao, Dong Liu, Jianguo Wang, Yaqi Lai, Yu Zhang

**Affiliations:** School of Materials Science and Engineering, Northwestern Polytechnical University, Xi’an 710072, Chinalaiyaqi2023@mail.nwpu.edu.cn (Y.L.); zy0429@mail.nwpu.edu.cn (Y.Z.)

**Keywords:** Ti6242, β forging, <111> fiber texture, metadynamic recrystallization, microtexture

## Abstract

The Ti-6242 titanium alloy samples were forged at 1020 °C (slightly above the β-transus) and subjected to ultra-short isothermal holding (0–320 s) prior to quenching to investigate the rapid microstructural evolution in the parent β phase. Electron backscatter diffraction (EBSD) with parent β-phase reconstruction reveals that within only 1–3 s of holding, a pronounced <111> fiber texture develops along the forging axis, superseding the original <100> deformation fiber. This ultrafast texture change is attributed to metadynamic recrystallization (MDRX)—the post-deformation growth of nuclei formed during dynamic deformation. The newly formed <111>-oriented β grains still contain residual substructure, indicating incomplete strain release consistent with MDRX. Longer holds (tens of seconds) lead to more extensive static recrystallization and normal grain growth, which dilute the strong <111> fiber as grains of other orientations form and coarsen. These findings demonstrate that even a brief pause after forging can markedly alter the prior β texture via a MDRX mechanism. This insight highlights a novel approach to microtexture control in Ti-6242: by leveraging MDRX during short holds, one can potentially disrupt the formation of aligned α colony microtextured regions (MTRs, or “macrozones”) upon subsequent cooling, thereby mitigating dwell-fatigue susceptibility. The study revises the interpretation of the recrystallization mechanism in short-term holds and provides guidance for optimizing β-phase processing to improve fatigue performance.

## 1. Introduction

Ti-6242, a typical near-alpha titanium alloy characterized by a microstructure predominantly composed of α phase with a minor fraction of retained β phase, is widely used in critical aerospace components owing to their excellent high-temperature strength and creep resistance [1,2]. However, this alloy can suffer from dwell-fatigue sensitivity, wherein aligned microtextured regions (MTRs, also called macrozones) of the α phase serve as sites for early crack initiation under dwell loading [3,4]. These MTRs arise from the β → α phase transformation; in other words, upon cooling, aligned lamellar α colonies precipitate from prior β grains and persist through subsequent processing [5,6]. Furthermore, for components produced by β forging or β annealing, the prior β grain characteristics (size and crystallographic texture) directly determine the transformed α-colony microtexture (e.g., MTR size and alignment) [7,8]. These microtextural features in turn strongly influence dwell-fatigue performance. Hence, mitigating such deleterious microtexture during β-phase processing is critical for improving the in-service reliability of Ti-6242 alloy.

Beta-phase thermomechanical processing is commonly employed to break up the as-cast microstructure and refine the prior β grain size. For Ti-6242 and similar alloys, β forging followed by controlled cooling can significantly alter the transformed α-phase microstructure and texture. The orientations of the prior β grains strongly influence which α variants form upon cooling, thereby controlling the degree of microtexture (colony alignment) in the α phase. For example, Zhao et al. [9] showed in a Ti-60 (5.6 Al, 3.7 Sn, 3.2 Zr, 0.5 Mo, 0.4 Nb, 1.0 Ta, 0.37 Si, 0.05 C, balance Ti) alloy that the transformed α texture was directly linked to the parent β texture via variant selection. Different hot-compression conditions changed the fractions of <111> β and <100> β fiber components in the prior β, which in turn altered the α colony orientations after cooling. Other researchers have demonstrated that adjusting the cooling rate after β deformation can modify variant selection and α-colony alignment [10,11]. Dong et al. [11] observed in a near-β Ti-55511 alloy that slower post-forging cooling (e.g., 0.05 °C/s vs. ≥0.1 °C/s) produced more extensive static recrystallization of β and refined β grains, thereby influencing the subsequent α variant texture. Together, these works underscore that both β deformation conditions and any subsequent thermal treatment (holding or cooling) are critical in determining the prior β texture and, consequently, the transformed α microtexture. By judiciously controlling the prior β grain size and texture through processing, one can aim to disrupt large aligned α colonies and thus minimize detrimental MTRs in the final microstructure.

Despite these insights, most prior studies have examined either continuous-cooling transformations or relatively long β-phase anneals (on the order of minutes to hours) [5]. The immediate post-deformation holding regime—on the order of only a few seconds—has received far less attention. This ultra-short hold regime is industrially relevant: even brief (unintentional) delays before quenching could alter the prior β microstructure and texture, potentially affecting the resulting α colonies and fatigue performance. Banerjee and Williams [2] have emphasized that advancing the predictive capability for titanium alloys requires a deeper understanding of phase transformation phenomena and microstructural instabilities during processing. In particular, the mechanisms of rapid recrystallization and texture change in the β phase during very short holds remain unclear. Notably, Pilchak et al. [12] observed in Ti-6Al-4V that a strong β deformation texture (a “rotated-cube” component) became even stronger during the early stages of β annealing, due to orientation-dependent recrystallization of the retained substructure. This suggests that significant texture evolution can occur on very short time scales in the β phase. However, such effects in near-α alloys like Ti-6242—especially any tendency to form specific texture components during ultra-short holds—have not been previously reported.

In this work, we address this knowledge gap by investigating the mechanisms of rapid texture change during ultra-short holds following β forging of Ti-6242. In particular, the formation of a pronounced <111> β fiber texture within mere seconds of holding was focused and identified metadynamic recrystallization (MDRX) as the governing mechanism. The findings provide new insights into how β texture can be controlled (or inadvertently altered) during β processing, with implications for designing thermomechanical treatments to minimize harmful MTRs in critical Ti-6242 components.

## 2. Materials and Methods

**Figure 1:**
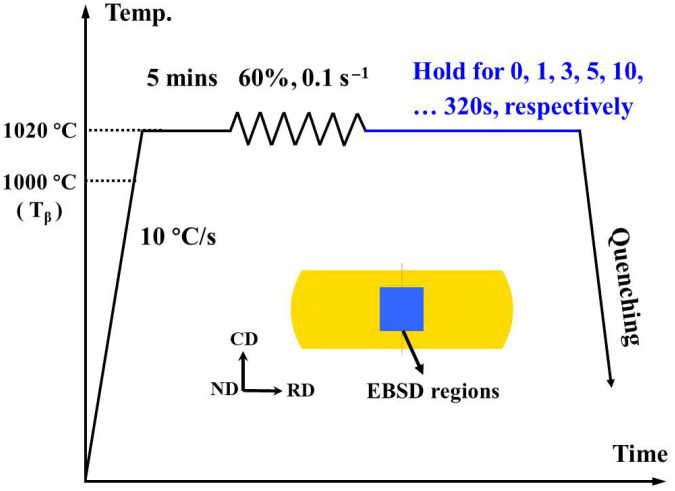
Schematic of the thermomechanical processing schedule applied to Ti-6242: solution heating into the β-phase field (5 min at ~1020 °C (~20 °C above the β-transus)), β forging (60% height reduction), followed by an isothermal hold at 1020 °C for 0~320 s and high-pressure jet quenching.

Bars of Ti-6242 (wt.%: 6.02 Al, 2.11 Sn, 4.14 Zr, 2.03 Mo, 0.12 O, 0.08 Si, 0.02 Fe, balance Ti) were used in this study. The β-transus temperature of this alloy is approximately 1000 °C. Thermomechanical processing was conducted using a Gleeble thermo-mechanical simulator (Gleeble, Poestenkill, NY, USA), following the route schematically illustrated in Figure 1. For β deformation, each sample was heated at 10 °C/s to 1020 °C (~20 °C above the β-transus) and held for 5 min to ensure a fully β-phase microstructure. The sample was then compressed (uniaxial forging) to 60% height reduction at a strain rate of 0.1 s^−1^. After deformation, each specimen was held isothermally at 1020 °C for a designated time (0, 1, 3, 5, 10, 20, 40, 80, 160, or 320 s) before being rapidly water-quenched to room temperature. The 0 s hold (immediate quench) provides a baseline as a deformed β structure with no post-deformation annealing, whereas the nonzero hold times simulate a short β anneal immediately after forging. To ensure the accuracy of the designated time, in the Gleeble test, after deformation, the sample is held for the specified time and then cooled using high-pressure jet cooling. For instance, a holding time of 0 s involves immediately cooling the sample with high-pressure jet cooling right after deformation.

Longitudinal sections (through the center of each Gleeble-processed sample) were prepared for microstructural analysis. Sections were ground and mechanically polished, followed by electropolishing in a solution of 6% perchloric acid + 33% butanol + 61% methanol at ~0 °C for ~40 s to produce a strain-free surface for electron backscatter diffraction (EBSD). Orientation mapping was performed using a field-emission SEM equipped with an Oxford Instruments EBSD detector (Oxfordshire, UK); large areas (several mm^2^) were scanned with a step size of 1 µm, achieving an indexing rate of over 95% (without the need for subsequent noise reduction processing). Since the room-temperature microstructure after quenching consists of α phase (with a small fraction of retained β), the prior β-phase orientations were reconstructed by applying the Burgers orientation relationship (BOR) to each α grain orientation. Groups of α variants corresponding to a common parent β grain were identified using the MATLAB (version R2020b) MTEX toolbox, thus allowing estimation of the prior β-grain structure and texture from the as-quenched α microstructure. For each condition, inverse pole figure (IPF) maps, grain orientation spread (GOS) maps, and pole figures (PFs) of the reconstructed β phase were generated to characterize the prior β microstructure and texture evolution.

## 3. Results

**Figure 2:**
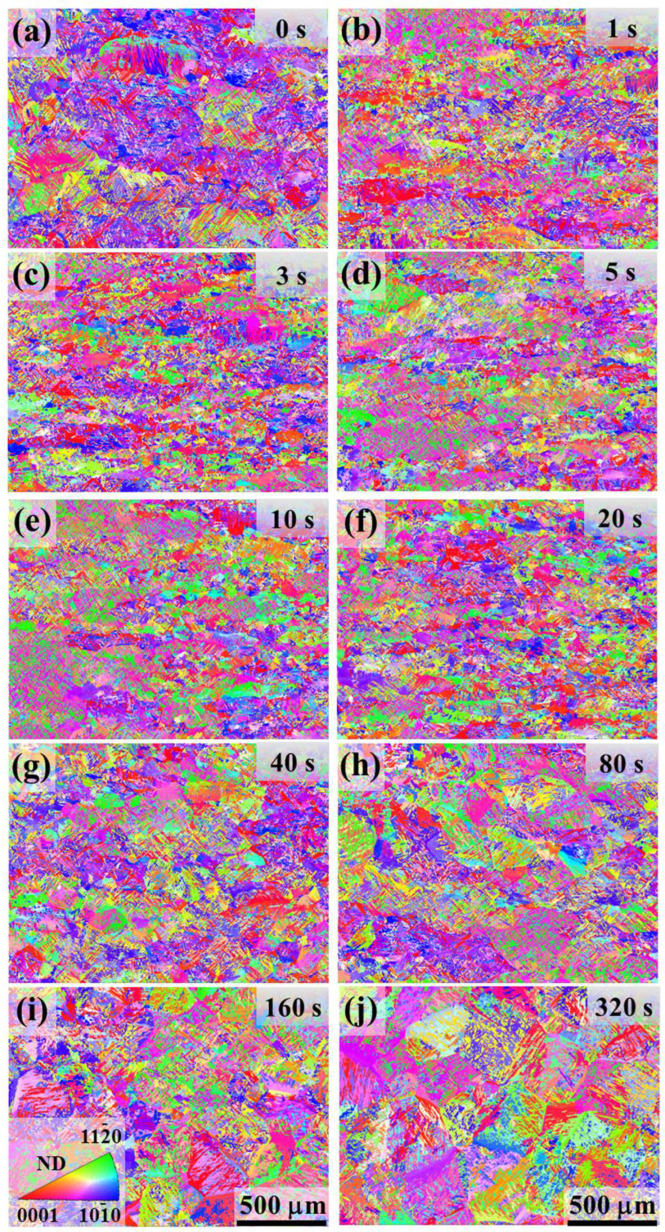
IPF maps for Ti-6242 after β forging followed by different hold times at 1020 °C: (**a**) 0 s, (**b**) 1 s, (**c**) 3 s, (**d**) 5 s, (**e**) 10 s, (**f**) 20 s, (**g**) 40 s, (**h**) 80 s, (**i**) 160 s, (**j**) 320 s.

**Figure 3:**
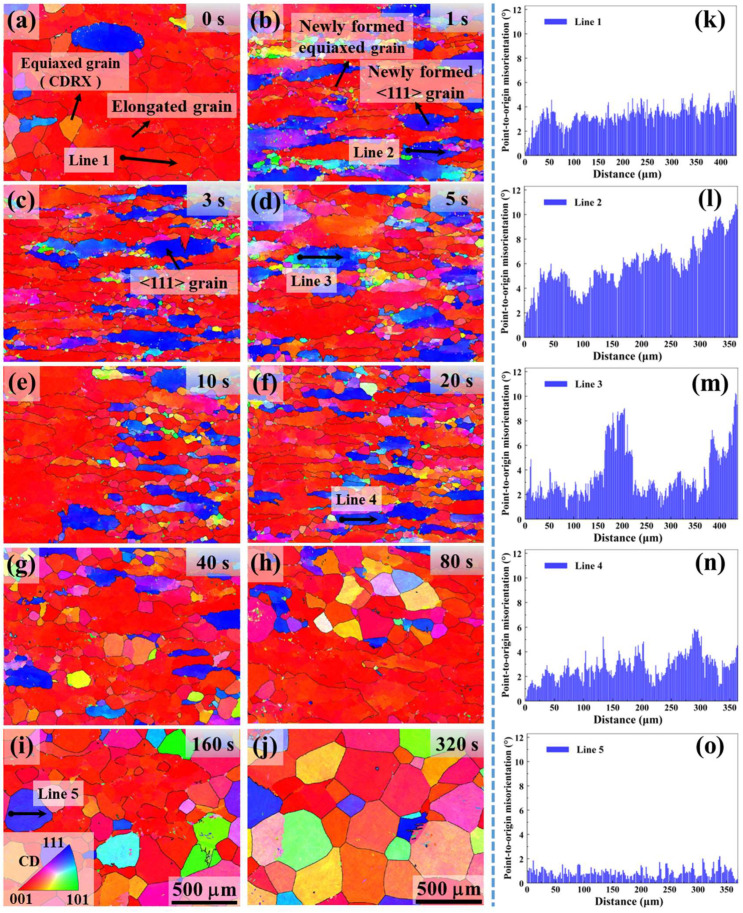
Reconstructed prior-β IPF maps for Ti-6242 after β forging followed by different hold times at 1020 °C: (**a**) 0 s, (**b**) 1 s, (**c**) 3 s, (**d**) 5 s, (**e**) 10 s, (**f**) 20 s, (**g**) 40 s, (**h**) 80 s, (**i**) 160 s, (**j**) 320 s. Panels (**k**–**o**) show point-to-origin misorientation distribution profiles (black arrows) across representative β grains for selected conditions: (**k**) 0 s, (**l**) 1 s, (**m**) 5 s, (**n**) 20 s, (**o**) 160 s. Black lines indicate high-angle grain boundaries (≥15°).

**Figure 4:**
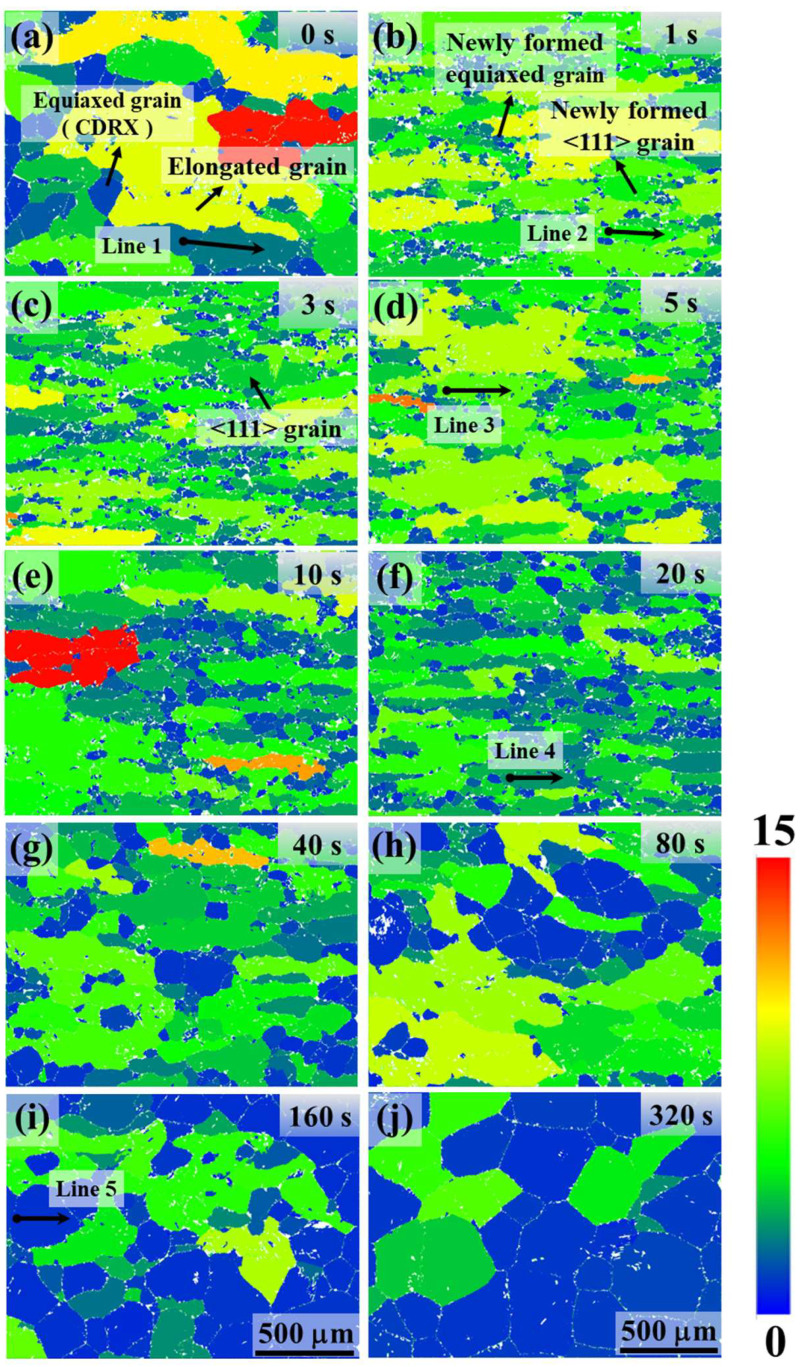
The corresponding GOS maps for the IPF maps in Figure 3, with (**a**–**j**) corresponding to the conditions in Figure 3a–j.

**Figure 5:**
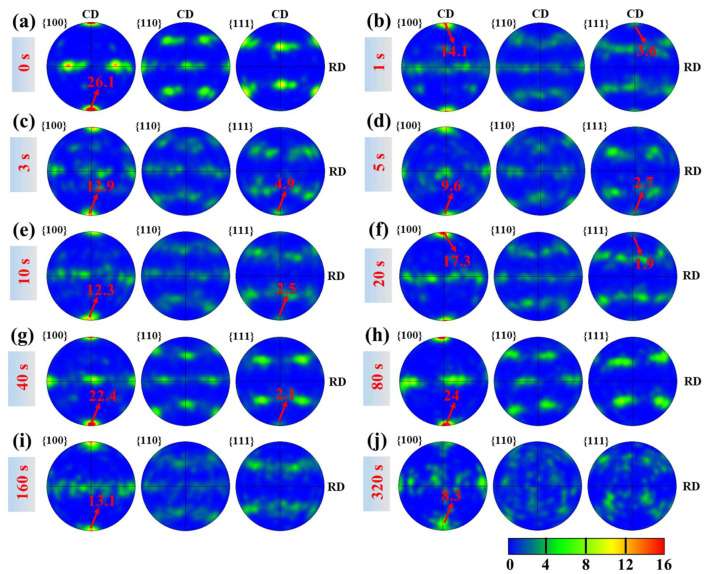
Pole figure maps for hold times from 0 to 320 s after β forging, with (**a**–**j**) corresponding to the conditions in Figure 3a–j.

Figure 2 shows EBSD inverse pole figure (IPF) orientation maps of the as-quenched microstructure for increasing hold times. After rapid quenching, all conditions exhibit a fully α-phase microstructure of fine lamellar α platelets inherited from the prior β grains. With increasing isothermal hold time before quenching, evidence of prior β-grain growth becomes apparent in the α microstructure (particularly for the longer holds, Figure 2g–j). For instance, by the 160 s and 320 s conditions, the as-quenched α plates form large regions of common orientation, consistent with the presence of very large prior β grains at those hold times. These observations provided the basis for reconstructing the prior β grain structure and analyzing β texture evolution. The reconstructed prior β grain maps are presented in Figure 3, with corresponding GOS maps and pole figures shown in Figure 4 and Figure 5.

Under the 0 s condition (no hold), the reconstructed prior β microstructure comprises large, elongated grains with a pronounced deformation fiber texture. Specifically, the prior β IPF map for 0 s (Figure 3a) shows a strong <100> fiber aligned with the forging axis, while the <111> component is negligible (shown in Figure 5a). Very few equiaxed β grains are evident at 0 s, indicating that dynamic recrystallization (DRX) during forging was minimal. Instead, the prior β grains contain a dense subgrain structure from dynamic recovery: many low-angle boundaries (LAGBs) are observed inside the elongated grains. A representative misorientation profile across a 0 s grain (Figure 3k) shows only a few degrees of cumulative misorientation from one end of the grain to the other, composed of numerous small misorientation steps (<5° each). This confirms that the interior of the forged β grains is partitioned into subgrains rather than new grains. Thus, immediately after forging, the prior β can be described as elongated, recovered β grains with extensive substructure and a strong <100>//forging texture.

Holding the forged sample even briefly at 1020 °C produces rapid microstructural changes. After just a 1 s hold, small equiaxed β grains (~30–50 µm) begin to appear along prior β grain boundaries and in regions of high local strain (e.g., near grain-boundary triple junctions or within shear bands) (Figure 3b). These nascent grains are delineated by high-angle grain boundaries (HAGBs ≥ 15°), indicating that recrystallization has occurred within only 1 s. Notably, many of the grains exhibit <111> orientations nearly parallel to the forging axis (appearing as blue regions in Figure 3b, since blue corresponds to <111>//axis). By 3 s of holding, the recrystallized fraction increases further (Figure 3c), indicating continued formation and growth of new grains. Accompanying this microstructural change is a striking texture transformation: within 3 s, a strong <111> fiber develops along the forging direction, effectively replacing the original <100> fiber. The {111} pole figure intensity at 3 s (Figure 5c) is dramatically higher than at 0 s (Figure 5a), whereas the <100> pole intensity drops. Thus, in the span of only a few seconds, the dominant crystallographic fiber in the prior β phase shifts from <100> to <111>.

Although the newly formed grains at 1–3 s are recrystallized in that they are bounded by HAGBs, they are not completely strain-free. The GOS maps (Figure 4b,c) show that these grains retain notable internal misorientation. Likewise, a misorientation line scan across a representative 1 s grain (Figure 3l) reveals on the order of 5–10° of internal misorientation from grain center to edge. This indicates that the 1–3 s grains still contain residual substructure and have not fully relaxed their stored deformation energy. As the hold duration increases beyond a few seconds, recrystallization (primarily the nucleation stage) becomes far more extensive. By 5 s and especially 20 s (Figure 3d,f), the fraction of equiaxed β grains continues to grow. These new grains span a variety of orientations. Consequently, the strong <111> fiber that peaked at 3 s begins to be diluted by the emergence of grains with other orientations. For example, the {111} pole figure intensity at 20 s (Figure 5f) is lower than at 3 s, and the poles are more widely spread, reflecting a more diverse texture. Consistently, the GOS values of grains at 10–20 s drop to very low levels (Figure 4e,f), indicating that by this time most grains have relieved their internal strain and become nearly strain-free.

During longer holds (tens to hundreds of seconds), the regular grain growth of the recrystallization process becomes the dominant mechanism. By 40–80 s, the recrystallized β grains begin to coarsen substantially (Figure 3g,h). At 160 s and 320 s (Figure 3i,j), the prior β microstructure consists of equiaxed grains that have grown to a few hundred microns in diameter on average. This grain growth further alters the texture: the initially sharp <111> fiber is progressively weakened as grains of various orientations coarsen. The pole figures for 40 s through 320 s (Figure 5g–j) show the <111> intensity decreasing relative to its peak at 3 s, with a correspondingly more diffuse orientation distribution. By 320 s, the prior β texture is considerably weaker and more random compared to the very pronounced fiber after short holds. The nearly fully recrystallized and grain-grown microstructure at 320 s exhibits negligible GOS (Figure 4j), confirming that it has reached a strain-free, annealed state.

## 4. Discussion

### 4.1. Ultrafast <111> Fiber Development via Metadynamic Recrystallization (0–3 s Holds)

After β-forging and immediate quenching (0 s hold), the prior β microstructure consists of large, elongated β grains aligned with their long axes roughly perpendicular to the compression axis (as expected for uniaxial forging). The prior β retains a strong <100>//forging-axis texture in this as-forged state, with essentially no new equiaxed β grains observed (indicating negligible DRX during deformation). This initial condition is consistent with the relatively high deformation temperature and the known behavior of β-titanium alloys: the β phase’s high stacking fault energy promotes recovery (subgrain formation) rather than substantial DRX at moderate strains [13,14,15]. Indeed, studies on a similar β-titanium alloy (TC18) have reported DRX fractions below ~10% during hot compression, with most of the strain accommodated by recovery [16]. As a result, the 0 s microstructure is characterized by a well-developed subgrain structure (numerous LAGBs within the elongated β grains) and a lack of new high-angle boundaries. The absence of significant HAGB formation by the end of forging suggests that the critical strain for widespread DRX was not reached. Thus, prior to any post-forging hold, the β structure can be described as elongated, recovered grains with a strong <100> fiber texture and internal subgrain networks—a state that provides the baseline for subsequent changes during holding.

During a short high-temperature hold of 1–3 s, the microstructure and texture evolve dramatically. New equiaxed β grains nucleate rapidly (primarily along prior β boundaries or in highly deformed regions) and a pronounced <111>//axis fiber texture develops, effectively superseding the original <100> deformation fiber. Evidently, these fine, newly formed, equiaxed grains exhibit typical static recrystallization (SRX) characteristics. The rapid emergence of a specific texture component is highly unusual in a post-deformation annealing context. Typically, SRX tends to weaken or randomize a deformation texture, since new grains often nucleate in a variety of orientations and reduce overall texture intensity. For example, annealing a cold-rolled β-titanium sheet was found to produce a much more random, weaker texture compared to the strong rolling texture. Here, however, the opposite in the initial seconds of holding was observed: the β texture sharpens in the <111> direction. The {111} poles concentrate strongly along the forging axis at 1–3 s (Figure 5), indicating that recrystallization in this early stage is orientation-selective—grains of certain orientations (<111>//forging) are preferentially forming and/or growing, rather than an orientation-agnostic, random nucleation of new grains. Such behavior points to a mechanism tied closely to the deformed substructure and its stored energy differences among orientations.

EBSD measurements of the new <111> grains’ substructure provide clues to this mechanism. The <111>-oriented β grains formed after 1–3 s still contain internal misorientations on the order of several degrees (Figure 3l and GOS in Figure 4b,c), whereas a fully, statically recrystallized grain that nucleated and grew as a strain-free crystallite would typically have negligible internal misorientation. The fact that the early-hold grains retain a substructure (multiple low-angle boundaries within each grain) suggests that they formed through a progressive rotation and amalgamation of subgrains, rather than by the classic discontinuous SRX involving nucleus formation and growth of a strain-free grain. In other words, the recrystallization mechanism operating in the first few seconds appears to be a continuous one (subgrain rotation recrystallization) akin to what has been termed continuous SRX in other contexts. Given the timing and circumstances (immediately post-deformation), it is appropriate to identify this mechanism as metadynamic recrystallization (MDRX) [17]. MDRX is defined as the rapid recrystallization that occurs immediately after deformation, utilizing the nuclei or high-misorientation subgrains that were generated during the deformation itself. In this case, although little DRX occurred during forging, the deformation created a dense network of subgrain boundaries (through dynamic recovery) and likely a few isolated high-angle boundaries at favorable sites (such as along prior β grain boundaries or within shear bands). Once the deformation ceased, these pre-existing “seeds” could quickly evolve into new grains by consuming surrounding subgrains using the stored energy difference. This post-deformation growth of deformation-induced subgrains is essentially the hallmark of MDRX.

The orientation-selective nature of this MDRX can be rationalized by considering stored energy and boundary mobility differences among orientations. In hot-worked metals (including β titanium), certain crystallographic orientations can accumulate higher dislocation densities or form more misoriented sub-boundaries during deformation, making them more prone to rapid recrystallization. Pilchak et al. observed in Ti-6Al-4V that subgrains with slight misorientations off the main texture component (i.e., orientations on the flank of a strong “rotated-cube” β texture) acted as preferred MDRX nuclei and grew at the expense of the primary orientation component [12]. In the Ti-6242 case, the initial deformation texture was <100>-fiber. The sudden rise in the <111> fiber within seconds suggests that subgrains of <111> orientation (perhaps those located at grain boundaries or within localized shear bands) had a stored energy or boundary mobility advantage, allowing them to grow rapidly and consume the surrounding matrix. In bcc β of titanium alloys, the <111> direction is the Burgers vector direction for the primary slip systems ({110}<111> and {112}<111>). Thus, β grains oriented with <111> roughly parallel to the compression axis likely activate multiple slip systems with high resolved shear stress, promoting a high density of dislocations and subgrain boundaries in those orientations. This could increase the stored deformation energy in <111>-oriented regions, making them energetically favorable sites for recrystallization. This kind of texture-controlled subgrain growth is strongly reminiscent of Pilchak’s observations [12]: the sequence of an initial texture sharpening (specific component growth) followed later by texture broadening is a signature of an MDRX-driven process.

By about 3 s of hold time, the metadynamic recrystallization has largely run its course in terms of generating <111>-oriented new grains. The <111> fiber intensity reaches its peak at ~3 s (Figure 5c). Essentially, a two-stage texture evolution was observed: an initial rapid stage (≤3 s) where orientation-selective MDRX produces a surge of <111>//axis grains (sharpening the <111> fiber), followed by a subsequent stage (>3 s) where more conventional SRX and grain growth processes take over (as discussed next).

**Figure 6:**
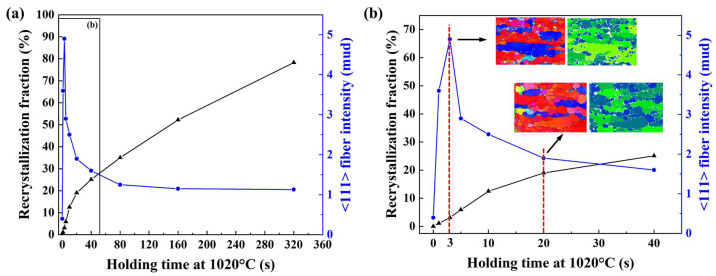
The recrystallization fraction and <111> fiber texture intensity evolution of β-forged Ti-6242 during the 1020 °C holding period: (**a**) 1~320 s period; (**b**) 1~40 s period.

For clarity, Figure 6 summarizes the kinetic characteristics of these processes. Figure 6a shows the curve of the recrystallized fraction of β grains (where grains with GOS values less than 1° are considered recrystallized grains free of internal stresses) as a function of holding time. During the initial stage of approximately 0–3 s, recrystallization progresses slowly. This stage is dominated by the nucleation of static recrystallization (SRX), resulting in a gradual increase in the recrystallized fraction. Although new MDRX grains are rapidly generated during this period, they still contain significant internal stresses. Subsequently, in the range of approximately 3–20 s, the recrystallized fraction increases at an accelerated rate, during which concurrent nucleation and growth of recrystallized nuclei occur. Beyond 20 s, the process is primarily governed by the growth of existing recrystallized nuclei. In the same figure, the intensity of the <111>//axial fiber texture component reaches its peak at approximately 3 s and then gradually decreases with prolonged holding time. This reveals a two-stage characteristic of microstructural evolution: in the initial stage, the combination of slow SRX progress and rapid MDRX action leads to the strengthening of the <111> fiber texture; in the later stage, the texture intensity gradually weakens as static recrystallization and grain growth proceed.

### 4.2. Extended Holds (≥10 s): Static Recrystallization, Grain Growth, and Texture Dilution

For hold times beyond a few seconds, the microstructure changes are governed by more conventional SRX and subsequent grain growth. By 10–20 s of holding, recrystallization nucleation is essentially complete—a part of the prior β has broken up into new equiaxed grains, resulting in substantial grain refinement (Figure 3e,f). By 160 s or 320 s, the microstructure has transformed into an equiaxed array of β grains that have started to coarsen via grain growth (Figure 3i,j). The prior β grain size after a long hold is on the order of hundreds of microns, significantly larger than the 10–50 µm grains observed at shorter holds, due to grain growth of the recrystallization.

As this longer-time SRX nucleation and grain growth proceed, the crystallographic texture gradually changes again. The sharp <111> fiber developed during the short hold is increasingly diluted by the formation and growth of grains with other orientations. Pole figures for 40 s, 80 s, up to 320 s (Figure 5g–j) show the <111> intensity decreasing relative to its peak at 3 s, while other orientation components become more pronounced. By ~20 s, the <111> fiber no longer intensifies; instead, general grain growth drives the texture towards a near-equilibrium state characterized by a low overall texture intensity by 160–320 s. Given sufficient time at temperature, recrystallization (especially if accompanied by some continuous growth of various orientations and perhaps secondary nucleation) and grain growth tend to weaken or randomize a prior deformation texture. For instance, prolonged anneals of cold-worked β-titanium produce textures that are much weaker and more random than the original deformation textures. The overall texture evolution we document—a specific texture component first strengthening, then later weakening—mirrors the two-stage behavior noted in classical studies of post-deformation annealing. Pilchak’s short-term β annealing experiments in Ti-6-4 [12], for example, reported an initial sharpening of a deformation texture component followed by its eventual reduction, attributing this to metadynamic recrystallization followed by standard static recrystallization and growth.

### 4.3. Implications for Microtexture Control and Dwell Fatigue

The above findings have important implications for microtexture control in Ti-6242 and similar alloys. Even an extremely short hold at the forging temperature (on the order of 1–3 s) can produce an “instant” recrystallization effect and significantly alter the orientation distribution of prior β grains. In practice, this means that brief, perhaps unintended, delays between deformation and quenching could introduce variability in the prior β microtexture and thus in the transformed α microstructure and mechanical performance. For instance, a hold of just a few seconds could trigger an MDRX-driven texture change that promotes certain α colony orientations upon cooling, whereas a slightly longer hold (tens of seconds) would result in a much finer, fully recrystallized β grain structure that transforms into a more random basketweave α colony structure. Recognizing this sensitivity, process engineers should account for any inter-step holds during forging and cooling: if not controlled, such holds may either mitigate or exacerbate the formation of aligned α colonies (macrozones) depending on their duration. On the other hand, this phenomenon can be exploited deliberately. By intentionally incorporating a brief hold at temperature under controlled conditions, one might tailor the prior β texture in a way that disrupts the continuity of deleterious α colonies. For example, promoting a strong <111>-fiber-dominated β texture via a short hold could scatter the orientations of subsequently formed α platelets (breaking up large microtextured regions), potentially reducing dwell-fatigue susceptibility. Conversely, ensuring either an immediate quench (to retain the deformation texture) or a sufficiently long hold (to fully recrystallize and refine the grains) could be used to achieve other desired microstructural outcomes. In essence, tight control of the post-forging cooling schedule—including even very short holds—emerges as a lever for optimizing microtexture.

From a process modeling and scheduling perspective, the results highlight the need to incorporate post-dynamic recrystallization effects into predictions of microstructural evolution. The occurrence of MDRX implies that sub-second or second-scale phenomena can have first-order effects on texture. Traditional static recrystallization models that ignore the influence of deformation-generated nuclei may underpredict the extent of recrystallization during short holds. Therefore, incorporating MDRX and continuous recrystallization mechanisms in simulation tools could improve the accuracy of microstructure predictions for near-α titanium alloys. In practical terms, if multiple forging steps are used, short inter-pass times (on the order of a few seconds) could lead to partial softening and texture changes between forging passes due to MDRX, which should be considered when designing forging schedules. Overall, being cognizant of these rapid post-deformation changes allows for better control (or avoidance) of certain microstructural states. By leveraging a brief hold to induce beneficial recrystallization or by minimizing dwell times to preserve a certain texture, one can refine the transformed α microtexture and potentially enhance the fatigue performance of critical Ti-6242 components.

Finally, while this study focused on Ti-6242, the principles are likely applicable to other titanium alloys and high-temperature materials. The interplay of metadynamic and static recrystallization is not unique to Ti-6242; prior observations in Ti-6Al-4V and in β-titanium models hint that similar ultrafast texture shifts can occur in other alloys when the conditions are right [12]. Thus, the findings contribute to the general understanding of microstructural evolution during thermomechanical processing, and they underscore the importance of controlling even very short thermal exposures in achieving desired final microtextures.

## 5. Conclusions

In this study, Ti-6Al-2Sn-4Zr-2Mo (Ti-6242) specimens were β-forged and subsequently subjected to ultra-short isothermal holding (0–320 s at 1020 °C), followed by quenching, to examine rapid microtexture evolution in the prior-β phase. The following conclusions were drawn:

(1) Ultrafast <111> fiber via MDRX: Brief holds (∼1–3 s) at 1020 °C after β-forging Ti-6242 trigger ultrafast development of a strong <111> fiber in the prior-β phase: numerous <111>//forging-axis β grains form via metadynamic recrystallization (MDRX) by progressive subgrain rotation, not classical nucleation/growth, promptly sharpening the <111> component and supplanting the initial <100> fiber.
(2) Effect of longer holds: With longer holds (tens–hundreds of seconds), static recrystallization and grain growth dominate. By ~10–20 s, the prior-β is largely recrystallized into fine equiaxed grains; at longer times, grain growth follows. Consequently, texture intensity declines—the initial <111> fiber is diluted—so by 320 s a weak, random texture and equiaxed microstructure remain.

## Data Availability

The data presented in this study are available on request from the corresponding authors due to privacy.
